# Direct peroral cholangioscopy with red dichromatic imaging 3 detected the perihilar margin of superficial papillary extension in a patient with intraductal papillary neoplasm of the bile duct

**DOI:** 10.1002/deo2.228

**Published:** 2023-03-27

**Authors:** Akinobu Koiwai, Morihisa Hirota, Keigo Murakami, Tomofumi Katayama, Ryo Kin, Katsuya Endo, Takayuki Kogure, Atsuko Takasu, Hiroto Sakurai, Noriko Kondo, Kazuhiro Takami, Kuniharu Yamamoto, Yu Katayose, Kennichi Satoh

**Affiliations:** ^1^ Division of Gastroenterology Tohoku Medical and Pharmaceutical University Miyagi Japan; ^2^ Division of Pathology Tohoku Medical and Pharmaceutical University Miyagi Japan; ^3^ Department of Investigative Pathology Tohoku University Graduate School of Medicine Miyagi Japan; ^4^ Division of Hepato‐biliary and Pancreatic Surgery Tohoku Medical and Pharmaceutical University Miyagi Japan

**Keywords:** endoscopic retrograde cholangiopancreatography, EVIS X1, intraductal papillary neoplasm of bile duct, peroral cholangioscopy, red dichromatic imaging

## Abstract

Intraductal papillary neoplasms of the bile duct (IPNB) are a tumor derived from bile duct epithelium that tends to spread laterally and non‐invasively. Surgery is the first‐choice treatment for IPNB. It is extremely important to accurately diagnose the extent of lateral tumor extension. Although peroral cholangioscopy (POCS) is a potentially useful modality for detecting tumor range with direct observation, poor image quality is a limitation of POCS. Recently, a new‐generation endoscopy system (EVIS X1) was equipped with functions such as red dichromatic imaging to improve image quality. A 75‐year‐old man with cholangitis was referred to our department. Various imaging studies showed a mass in the middle to lower bile duct and dilatation of the common bile duct and the intrahepatic bile duct. Endoscopic retrograde cholangiopancreatography was performed. A biopsy of the main tumor in the lower common bile duct revealed IPNB. It was difficult to determine the extent of superficial tumor extension with modalities such as contrast‐enhanced computed tomography, magnetic resonance imaging, and endoscopic ultrasonography but the detailed evaluation was possible using POCS with red dichromatic imaging 3. The patient underwent hepatopancreatoduodenectomy. This case suggests the usefulness of direct observation using POCS with red dichromatic imaging 3 to determine the range of IPNB.

## INTRODUCTION

Intraductal papillary neoplasm of the bile duct (IPNB) is an epithelial tumor characterized by dilated intrahepatic and extrahepatic bile ducts filled with papillary biliary tumors covering thin fibrovascular stalks.^1^, ^2^ IPNB is pathologically classified into two types, type 1 and type 2. Type 1 is histologically similar to intraductal papillary mucinous neoplasm of the pancreas; it typically develops in the intrahepatic bile ducts. Type 2 has a more complex histological architecture with irregular papillary branching or foci of solid tubular components; it typically involves the extrahepatic bile ducts.^3^ For both types of IPNB, surgery is the first‐choice treatment. IPNB has a relatively better prognosis after resection.^4^ It is extremely important to accurately diagnose the range of intraepithelial extension because it has a tendency to infiltrate and extend into the epithelium of the bile ducts in the longitudinal direction.^1^

Peroral cholangioscopy (POCS) can visualize the bile ducts, allowing for assessment of the surfaces and other characteristics of intraductal tumors and the surrounding biliary mucosa.^5^, ^6^ Recently, a new‐generation endoscopy system from Olympus Marketing (Tokyo, Japan), EVIS X1, has become commercially available. EVIS X1 is equipped with image enhancement functions such as red dichromatic imaging (RDI) in addition to better image quality. RDI has three modes (RDI1–3) for different intended uses. Previously, we reported on the usefulness of RDI3 in POCS.^7^ Here, we report a case of IPNB in which accurate identification of tumor margins in the perihilar region was made with a preoperative POCS examination using RDI3.

## CASE REPORT

**FIGURE 1:**
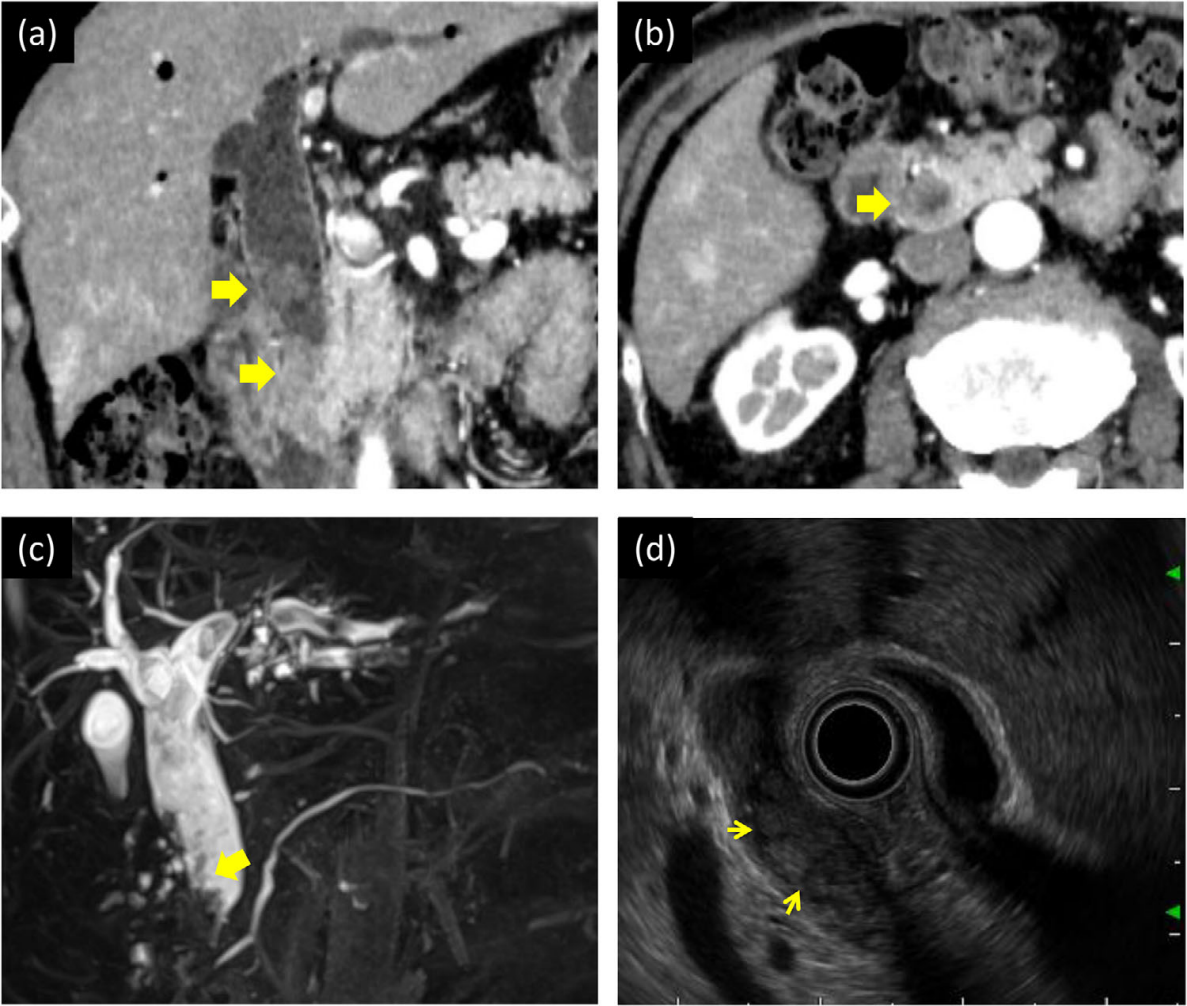
Imaging findings of the mass with low enhancement (yellow arrows), which occupied the middle to lower bile duct, and dilatation of the common bile duct and intrahepatic bile ducts. Coronal (a) and axial (b) abdominal contrast‐enhanced computed tomography images. (c) On magnetic resonance cholangiopancreatography, a tumor‐like defect in the common bile duct (yellow arrow) with dilatation of the intrahepatic and common bile ducts was observed. (d) Endoscopic ultrasonography revealed dilatation of the common bile duct, the elevated papillary tumor in the middle bile duct (yellow arrow), and mucus accumulation in the upper bile duct. The proximal bile duct wall was mildly thickened. The hilar bile ducts could not be fully observed.

**FIGURE 2:**
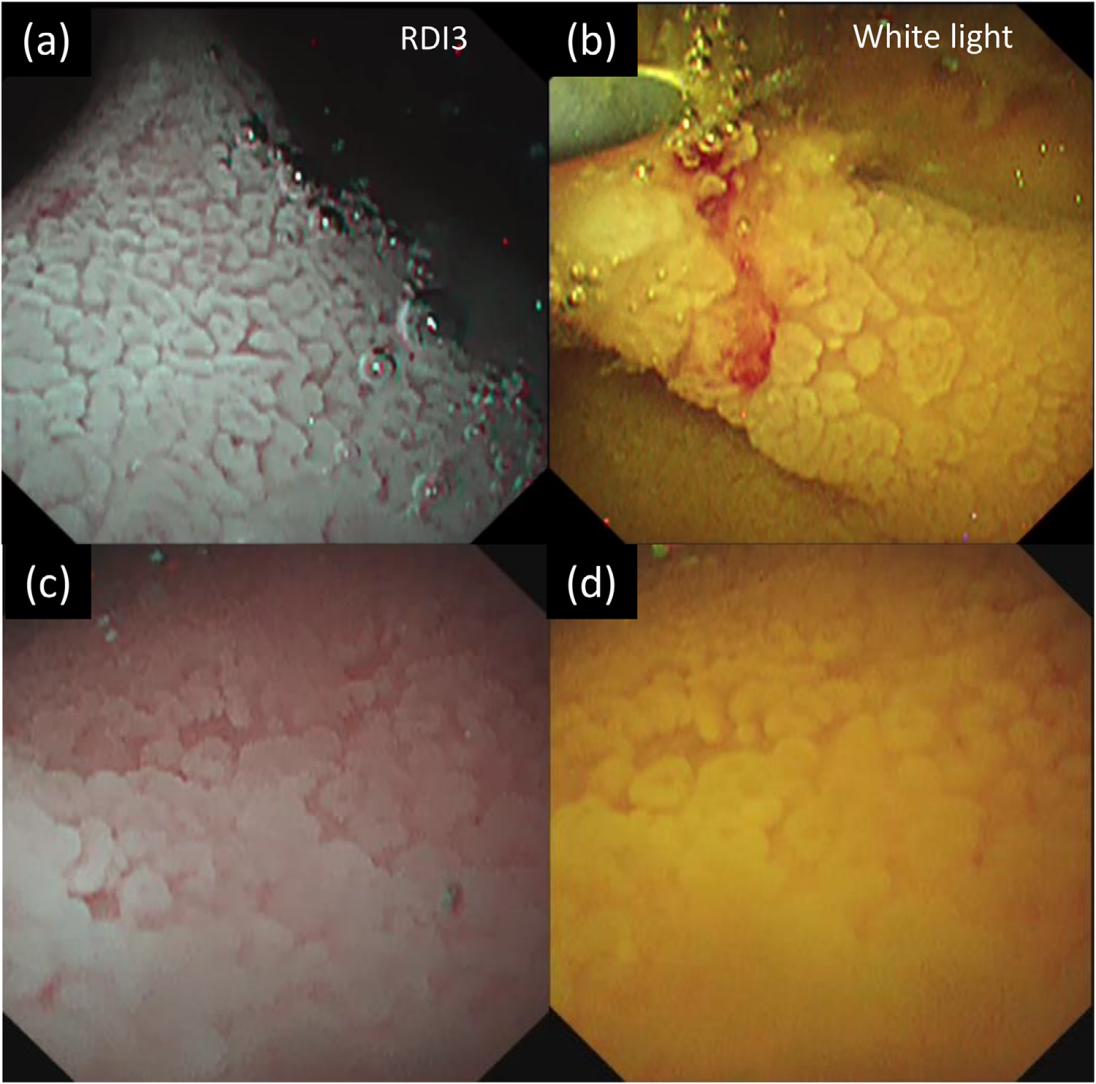
Slightly elevated papillary tumor located in the hilar bile ducts was observed using peroral cholangioscopy with red dichromatic imaging 3 (a) or with white light imaging (b), and a papillary tumor in the upper bile ducts was observed using peroral cholangioscopy with red dichromatic imaging 3 (c) or with white light imaging (d) in close observation.

**FIGURE 3:**
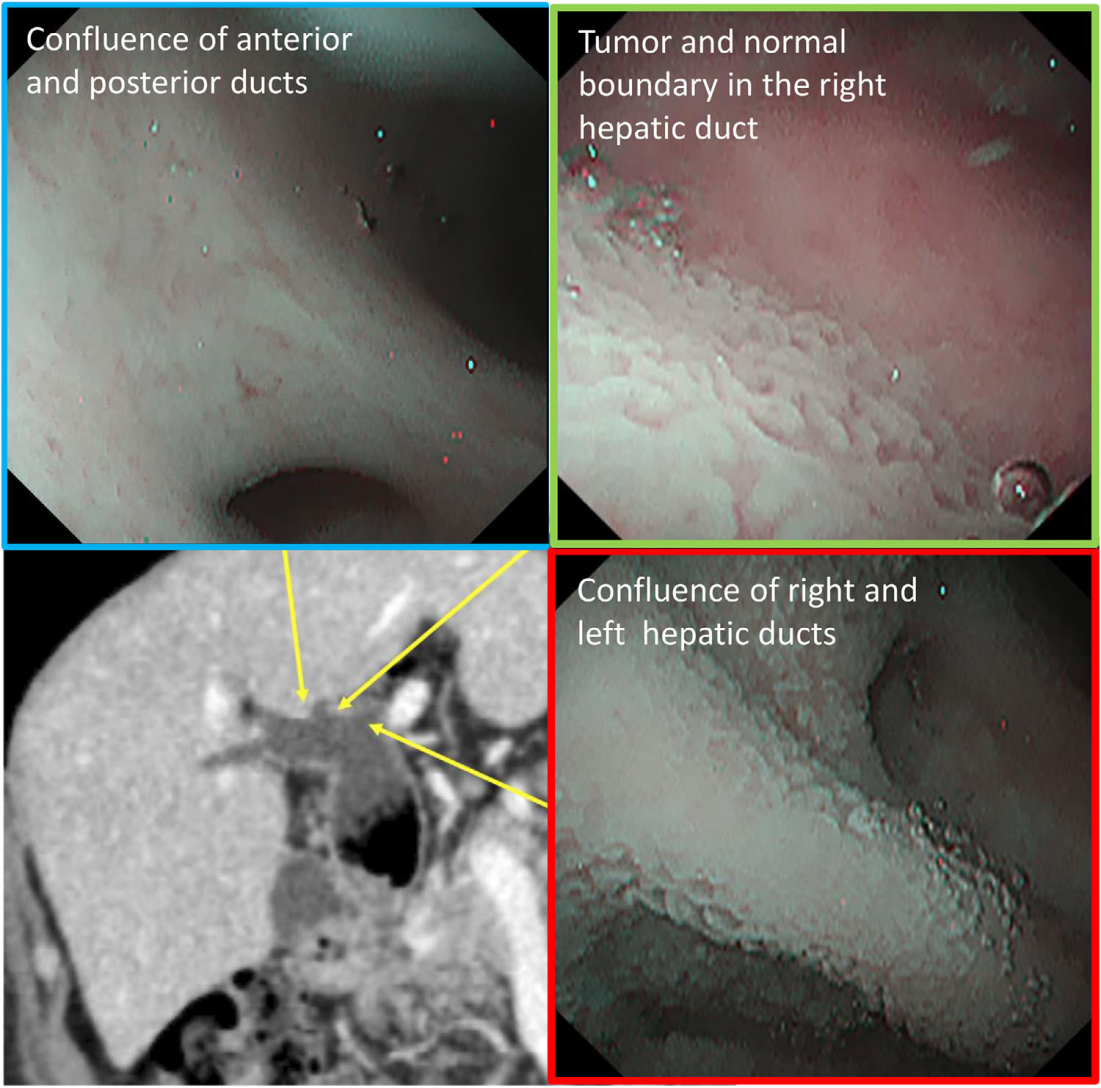
The contrast diagram of contrast‐enhanced computed tomography findings and peroral cholangioscopy with red dichromatic imaging 3. Images of peroral cholangioscopy with red dichromatic imaging 3; the confluence of the right anterior and right posterior segmental bile duct, tumor and normal boundary in the right hepatic duct, the confluence of the right and left hepatic ducts. The red frame indicates intraductal papillary neoplasm of the bile duct (high grade), the Green frame indicates intraductal papillary neoplasm of the bile duct (low grade), and the blue frame indicate normal mucosa.

**FIGURE 4:**
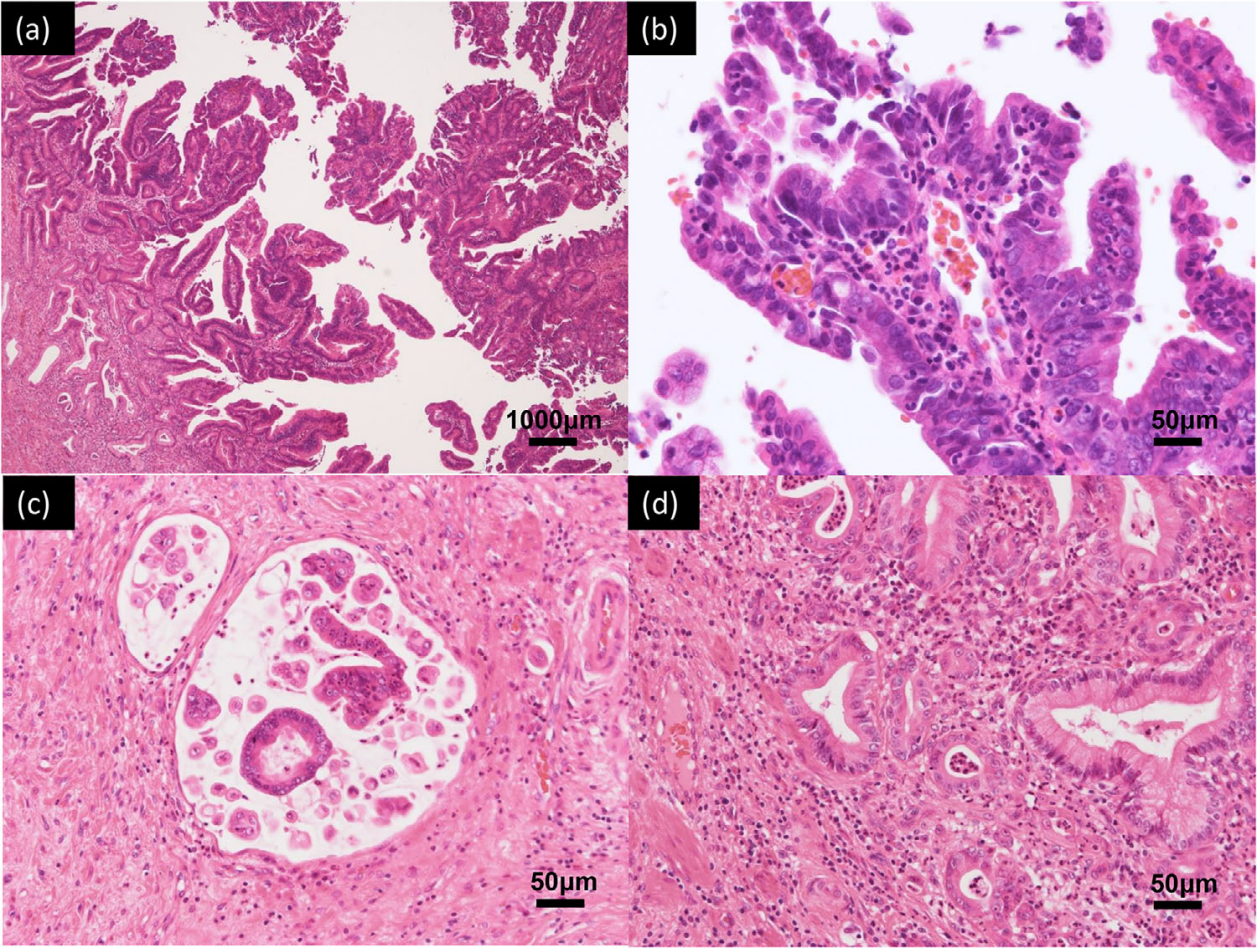
Pathological findings from intraductal papillary neoplasm of the bile duct. (a) A papillary tumor with a thin fibrous vascular stalk was observed throughout almost the entire excised extrahepatic bile duct (hematoxylin‐eosin staining). (b) High‐power view of the papillary tumor with high‐grade dysplasia (hematoxylin‐eosin staining). The distal bile duct had invasive carcinoma, which was equivalent to mucinous adenocarcinoma (c) > well‐differentiated type (d) (hematoxylin‐eosin staining).

A 75‐year‐old man was admitted to another hospital with complaints of upper abdominal pain and fever. He was diagnosed with cholangitis and treated with antibiotics. He was referred to our department for further examination and treatment. He had hypertension and a history of cholangitis more than 10 years ago; the details were unavailable. Laboratory tests showed elevated values of alkaline phosphatase, 114 U/L; γ‐glutamyl transpeptidase, 147 U/L; and carbohydrate antigen 19‐9, 63.5 U/ml. Levels of total bilirubin, aspartate aminotransferase, alanine aminotransferase, and other tumor markers (carcinoembryonic antigen, S‐pancreas‐1 antigen, and duke pancreatic monoclonal antigen type‐2) were within the normal range. Contrast‐enhanced computed tomography revealed a tumor with slight enhancement on the luminal side of the middle to lower bile duct as well as dilatation of the common bile duct and intrahepatic bile ducts (Figure 1a,b). No nodal or distant metastases were identified. In addition, no wall thickening or masses in the hilar bile ducts or intrahepatic bile ducts were detected (Figure 1a). Magnetic resonance cholangiopancreatography showed a tumor‐like defect in the common bile duct with dilatation of the intrahepatic and common bile ducts (Figure 1c). Endoscopic ultrasonography (EUS) from the duodenal bulb revealed dilatation of the common bile duct, the elevated papillary tumor detected in the middle to lower bile duct, and mucus accumulation in the upper bile duct (Figure 1d). The tumor did not have findings suggestive of pancreatic invasion. The wall of the proximal bile duct was mildly thickened. Although we consider the possibility of lateral extension, the hilar bile ducts could not be fully observed with EUS. Endoscopic retrograde cholangiopancreatography revealed a papillary‐shaped defect in the bile duct. Subsequently, POCS using CHF‐B290 (Olympus Marketing) and EVIS X1 showed the elevated papillary tumor occupying the lower to the middle bile duct. Since detailed observation was difficult because the tumor was covered with a large amount of mucus, a biopsy was performed. Histological examination revealed high‐grade dysplasia. POCS focused on the hepatic perihilar region because it was important to determine the tumor margins for operability. Granular changes were observed from the common bile duct to the hilar bile ducts. Low papillary elevations on the bile duct surface were more clearly observed with RDI3 than with white light imaging (Figure 2a,b). Slight mucosal irregularities could be clearly observed with RDI3 compared to white light imaging, even at close proximity. (Figure 2c,d). Tumor extension to the confluence of the left and right hepatic ducts was observed. The boundary between the tumor and normal epithelium was found in the right hepatic duct with RDI3 (Figure 3). No granular changes were observed at the confluence of the right anterior and right posterior segmental ducts (Figure 3). Biopsy was performed during POCS at the B4 confluence, at the confluence of the right anterior and right posterior segmental ducts, and at the confluence of the left and right hepatic ducts. The specimen from the confluence of the right anterior and right posterior segmental ducts consisted of non‐neoplastic mucosa. Other specimens consisted of IPNB with high‐grade dysplasia. Biopsy results supported the observations during POCS. Hepatopancreatoduodenectomy was performed. The surgical margins at the right hepatic duct were tumor‐free, as previously diagnosed by POCS. The patient was discharged without any problems after the operation. Histologically, papillary tumor cells with a thin fibrous vascular stalk were observed over almost the entire excised extrahepatic bile duct (Figure 4a,b). The tumor was diagnosed as type 2 IPNB with associated microinvasion, which was not evident on imaging (Figure 4c,d). Immunostaining results (mucin (MUC)1 (+), MUC2 (−≫+), MUC5AC (+≫−), MUC6 (+≫−)) and tumor morphology suggested pancreatobiliary‐type IPNB.

## DISCUSSION

IPNB, a precursor of cholangiocarcinoma, is similar to intraductal papillary mucinous neoplasm of the pancreas but has a higher malignant transformation rate than intraductal papillary mucinous neoplasm.^8^ Surgery is the only curative treatment for IPNB. IPNB tends to extend horizontally along the bile duct wall. The extent of the lateral extension needs to be carefully investigated to determine whether surgery is possible. Several diagnostic imaging modalities, such as endoscopic retrograde cholangiopancreatography, POCS, EUS, intraductal ultrasonography (IDUS), contrast‐enhanced computed tomography, and magnetic resonance cholangiopancreatography are useful for the preoperative evaluation of bile duct tumors. The diagnosis of intraepithelial extension, as often seen in IPNB, is relatively difficult. Most cases can be diagnosed with EUS and IDUS. However, it is still difficult to identify intraepithelial extension when the epithelial changes are minor. POCS might lead to a proper diagnosis of intraepithelial extension.^9^, ^10^

Previously, we reported on the usefulness of RDI3 in POCS.^7^ RDI3 made it possible to obtain clearer images of the fine surface structure and the small vessels of the mucosa despite the presence of yellow bile compared with white light observation. RDI3 provided images of higher quality, resulting in the ability to observe clearly and to target biopsy accurately. In this case, IPNB was mainly located in the lower bile ducts but extended superficially to the hilar bile ducts. Despite the slightly elevated papillary lesions, the extent of lateral IPNB spread could be evaluated in detail using POCS with RDI3. IPNB has a better prognosis than conventional cholangiocarcinoma. Thus, accurate preoperative diagnosis of the extent of lateral spread is important to improve the curative resection rate. POCS with RDI3 will allow us to observe more detail than conventional POCS. Currently, it was not possible to evaluate the deep blood vessels, but the deep blood vessels can be emphasized and visually recognized with RDI3. In the future, detailed observation of such deep blood vessels might enable endoscopic classification with POCS. We hope that POCS observation with RDI3 will be useful for differentiating between inflammatory and neoplastic diseases of the bile ducts in the future.

## CONFLICT OF INTEREST STATEMENT

None.
